# Extracellular pH, cell length and cell differentiation do not firmly correlate across *Arabidopsis* root tissues

**DOI:** 10.1093/pcp/pcaf031

**Published:** 2025-03-24

**Authors:** H. Nicholay Diaz-Ardila, Christian S Hardtke

**Affiliations:** Department of Plant Molecular Biology, Biophore Bldg., University of Lausanne, CH-1015 Lausanne, Switzerland; Department of Plant Molecular Biology, Biophore Bldg., University of Lausanne, CH-1015 Lausanne, Switzerland

The Acid Growth Theory (Rayle and Cleland 1970) posits that cell wall acidification stimulates plant cell elongation. However, to what degree this applies to root cells remains a subject of debate. Here, we simultaneously monitored a sensor of apoplastic pH, a marker of cell differentiation, and cell length as a proxy of elongation to investigate the correlation of these parameters across *Arabidopsis* root tissues. Our measurements suggest that, with the possible exception of the epidermis, extracellular pH (pH_e_) generally does not limit cell elongation in the root.

Stem cell daughter cells divide repeatedly before they expand and differentiate. This is easily observed in the *Arabidopsis* (*Arabidopsis thaliana*) root apical meristem, where different cell layers undergo distinct developmental trajectories. For instance, whereas protophloem sieve element (PPSE) precursors only undergo a few divisions before elongation–differentiation, precursors of epidermal tissues divide more frequently and transition to elongation–differentiation later. The amended Acid Growth Theory suggests that cell wall acidification promotes cell growth because a low pH activates expansins, secreted proteins that promote cell wall loosening (Cosgrove 2005, Arsuffi and Braybrook 2018), and that auxin stimulates proton extrusion, as shown in hypocotyl elongation (Takahashi et al. 2012, Spartz et al. 2014, Fendrych et al. 2016, Lin et al. 2021). Yet, while auxin treatment generally triggers extracellular acidification in the shoot, it can also promote rapid extracellular alkalinization in the root, slower acidification notwithstanding (Fendrych et al. 2016, 2018, Li et al. 2021, Dubey et al. 2023, Serre et al. 2023). Moreover, increased steady-state auxin levels do not necessarily result in lower pH_e_ (Pacheco-Villalobos et al. 2016), and an auxin minimum may trigger the transition from cell proliferation to elongation (Pacifici et al. 2018). Finally, gradual pH_e_ alkalinization rather than acidification is associated with elongation–differentiation of PPSEs (Diaz-Ardila et al. 2023). Thus, whether the Acid Growth Theory is universally applicable to root tissues remains unclear.

The study of pH_e_ has used different methodologies like microelectrodes, pH-sensitive fluorescent dyes, and fluorescent proteins (Gjetting et al. 2012, Pacheco-Villalobos et al. 2016, Barbez et al. 2017, Martiniere et al. 2018, Moreau et al. 2022). While studies using pH-sensitive fluorescent dyes were largely restricted to observations of the root surface and outer tissue layers, genetically encoded pH sensors also permit pH_e_ estimation in inner tissues at cellular resolution. The ratiometric sensor PM-Apo-acidin4 permits noninvasive pH_e_ monitoring near the plasma membrane, where plant cells sense pH_e_ conditions (Martiniere et al. 2018, Liu et al. 2022). PM-Apo-acidin4 consists of fluorescent proteins with different pH sensitivities, expressed using the constitutive *35S* promoter. The fluorescence intensity ratio between 488 and 561 nm excitation in a plasma-membrane-adjacent region of interest is a proxy for pH_e_, with a lower ratio indicating more acidic conditions (Martiniere et al. 2018, Moreau et al. 2022) (Supplementary Fig. S1). We combined PM-Apo-acidin4 with a marker of cell differentiation, MINIYO (IYO) (Sanmartin et al. 2011, Munoz et al. 2017). In undifferentiated cells, IYO-GFP fusion protein is localized in the cytoplasm and barely detectable. However, at the onset of differentiation, it accumulates in the nucleus (Sanmartin et al. 2011). Together with morphological measurements, we could thus compare pH_e_ with cell elongation–differentiation across root tissues.

We previously reported that PPSE development involves pH_e_ alkalinization (Diaz-Ardila et al. 2023), which was not observed in adjacent cell files at equivalent distances from the stem cell niche (Supplementary Fig. S2a and b). Compared to developing protophloem, pH_e_ is, for example, more acidic in the stem cell niche (Supplementary Fig. S3a, c, e, and f). When grown on media buffered at pH7.5 rather than the standard pH5.7, both PPSEs and the stem cell niche displayed a more alkaline pH_e_ throughout (Supplementary Fig. S3b, d, e, and f). Transfer of roots on media buffered at different pH allowed us to gauge the robustness of the root apoplastic spaces to outside pH fluctuation. Generally, an initially strong response was observed after short transfers but dampened upon longer exposure (Supplementary Fig. S4a–g), although an overall higher apoplastic pH persisted upon prolonged growth on pH7.5 (Supplementary Fig. S4e–g). Thus, consistent with previous findings (Martiniere et al. 2018), roots can counteract pH_e_ fluctuations in the rhizosphere to some degree, for example, by adjusting plasma membrane H^+^-ATPase activity.

Simultaneous PM-Apo-acidin4 and IYO-GFP imaging confirmed the coincidence of PPSE elongation with the onset of differentiation and apoplastic alkalinization (Supplementary  Fig. 1a–h). Unfortunately, we were not able to follow other single vascular tissue cell files from initiation to elongation–differentiation because compared to PPSEs their differentiation occurs much later (Graeff and Hardtke 2021). Comprehensive monitoring of cell length, pH_e_, and IYO-GFP dynamics was however possible for the epidermis, cortex, endodermis, and pericycle, where we could follow individual cell files from their stem cells to their differentiated descendants in multiple roots and thereby obtain quantitatively robust data. In the pH_e_ measurements, we obtained similar trends for the periclinal (Supplementary Fig. S5b–f) and anticlinal cell walls (Fig. 1j–n). However, we primarily considered the anticlinal walls between subsequent cells for our conclusions because they were less prone to signal interference from neighboring cell files. Moreover, while cell elongation is an easily visible morphological feature linked to differentiation (Supplementary Fig. S6), it is less obvious in the distal tissues, such as the columella and lateral root cap. Nuclear IYO-GFP accumulation suggests that they differentiate rapidly after very few stem cell daughter divisions (Supplementary Fig. S6a). Interestingly, coincident extracellular alkalinization and differentiation were obvious in the lateral root cap (Fig. 1i). Since both columella/root cap cells and PPSEs undergo controlled autophagy (Furuta et al. 2014, Huysmans et al. 2018), it appears possible that extracellular alkalinization may be a feature of this process. Unlike in the protophloem and columella/root cap, alkalinization was not observed in any other tissues. Rather, gradual acidification was observed in epidermal cell files, whereas pH_e_ was essentially stable in cortex, endodermis, and pericycle cell files (Fig. 1j–n). Thus, except in the epidermis, we observed no strong correlation between cell elongation and pH_e_. We also observed that across cell layers at the same region where protophloem differentiates, the protophloem and epidermis have a higher pH_e_ in comparison with the other tissues (Supplementary Fig. S5a), corroborating a similar trend previously reported for mature root cells (Martiniere et al. 2018). Moreover, we could not observe any nuclear IYO-GFP accumulation in pericycle cells (Fig. 1n), even in mature parts of the root. This reiterates that cell elongation is not always firmly associated with cell differentiation as previously reported for the metaphloem (Graeff and Hardtke 2021), but it is also consistent with the reported maintenance of cell division capacity in pericycle cells and the idea that they constitute an extended stem cell pool along the root (Beeckman and De Smet 2014, Zhang et al. 2022).

**Figure 1.**
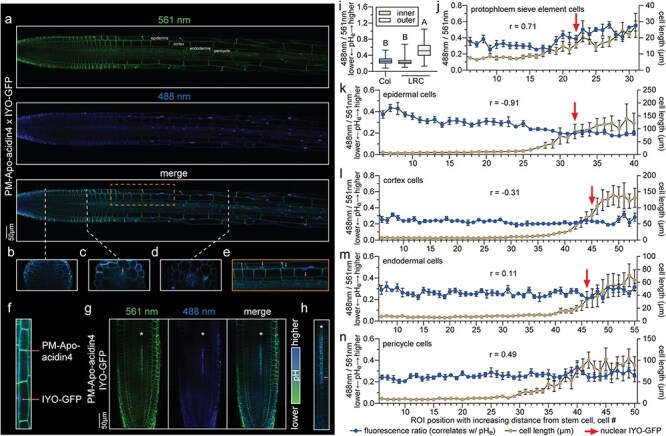
Simultaneous assessment of pH_e_, cell elongation, and cell differentiation in different tissues of *Arabidopsis* root tips. (a) Confocal live imaging of PM-Apo-acidin4 and IYO-GFP in the double-reporter line *35S::PM-Apo-acidin4 × 35S::IYO-GFP* in Col-0, 7-day-old seedlings. (b–d) Cross-sectional views of the indicated zone in (a), the arrowhead highlights the protophloem. (e) Close-up on the indicated zone in (a), with arrowheads indicating the nuclear signal of IYO-GFP in the epidermis, cortex, and endodermis from outside to inside. (f) Close-up view of cortical cells showing plasma membrane fluorescence of PM-Apo-acidin4 and the nuclear signal of IYO-GFP in the double reporter line *35S::PM-Apo-acidin4 × 35S::IYO-GFP*. (g) Confocal live imaging in root apical meristems of *35S::PM-Apo-acidin4 × 35S::IYO-GFP* in 7-day-old seedlings. Asterisks point out the protophloem cell file. (h) Close-up of the developing protophloem in the merged image of (g), the arrowhead marks the appearance of the nuclear signal of IYO-GFP. Asterisk points out the protophloem cell file. (i) Quantification of PM-Apo-acidin4 fluorescence in the columella and lateral root cap (inner and outer cells) of 7-day-old seedlings in the *35S::PM-Apo-acidin4 × 35S::IYO-GFP* line. Box plots display second and third quartiles and the median, and whiskers indicate maximum and minimum. Statistically significant differences were determined by ordinary one-way ANOVA (*n* = 8). (j–n) Anticlinal ratio-metric quantification of PM-Apo-acidin4 fluorescence, cell length, and IYO-GFP nuclear appearance in the protophloem (j), epidermis (k), cortex (l), endodermis (m), and pericycle (n) of 7-day-old seedlings. Spearman’s *r* is indicated for the correlation between the pH_e_ and cell length measurements. Red arrows indicate the nuclear appearance of IYO-GFP. Error bars indicate the standard error of the mean (*n* = 8).

In summary, our simultaneous observation of pH_e_, onset of differentiation, and cell length suggests that cell wall acidification generally does not limit the growth of root tissues, with the possible exception of the epidermis. This may reflect the close contact of roots with the surrounding soil environment, which could impose environmental constraints on pH_e_ regulation that are not experienced by shoot tissues. If root cells indeed typically maintain optimal pH_e_ conditions for elongation, the differential expression of effector proteins such as expansins may become limiting instead.

## Supplementary Material

pcaf031_Supp

## Data Availability

This study does not have data deposited in external repositories.
