# Supplementary material for: Extracellular pH, cell length and cell differentiation do not firmly correlate across Arabidopsis root tissues
Source: Plant Cell Physiol. 2025 Mar 24;66(6):836–9. doi: 10.1093/pcp/pcaf031 (PMC12290282; doi:10.1093/pcp/pcaf031)
Supplement: pcaf031_Supp [file pcaf031_supp.zip › suppl_data/pcp-2025-e-00010-File003.pdf]

MATERIAL AND METHODS

Plant material and growth conditions.

The study used the *Arabidopsis thaliana* accession Columbia-0 (Col-0) as the wild-type background for all experimental lines. For tissue culture, seeds were surfaced sterilized and subsequently sown on half-strength Murashige and Skoog medium (MS) supplemented vitamins, MES buffer, (Duchefa Product No. M0255), 0.3% sucrose, and 1% bacto-agar. For liquid culture, the same reagents and concentrations were used, excluding bacto-agar. The pH of the media was adjusted with KOH prior to adding agar for solid media and autoclaving. Seeds were stratified in darkness at 4°C for 3 days, then vertically placed in a growth chamber at 22°C under continuous light with a light intensity of ca. 120-130 μmol $m^{-2}s^{-1}$.

Transgenic lines.

The *PM-Apo-acidin4* and *35S::IYO-GFP* transgenic lines have been previously described (Moreau et al., 2022; Sanmartín et al., 2011). The double reporter line, *PM-Apo-acidin4 35S::IYO-GFP*, was generated by crossing.

pH treatments.

Seeds were plated on standard MS (pH5.7) and alkaline media (pH 7.5) and grown for seven days before confocal imaging. For the 1-hour treatment, seedlings initially grown on standard medium were transferred to alkaline medium (pH 7.5) for one hour, after which they were observed and imaged using confocal microscopy.

In vivo calibration of PM-Apo-acidin4 in Arabidopsis root tips.

Seedlings were grown under standard medium conditions for seven days before being transferred to either liquid or solid MS medium. Imaging was conducted as quickly as possible to minimize buffering of the extracellular pH by the plants. The fluorescence intensities were measured at the outer face of the epidermal cells, as these cells are in direct contact with the medium.

Microscopy and image analysis.

Confocal imaging was conducted using the Leica Stellaris 5 inverted confocal microscope. Fluorescent proteins and staining signals were visualized with the following emission-excitation wavelengths: GFP: excitation 488nm, emission 493-538 nm and propidium iodide (PI): excitation 514 nm, emission 589 – 725 nm. Roots were prepared for microscopy either in live imaging or stained with PI as described (Graeff and Hardtke, 2021).

The pH sensors *PM-Apo-acidin4* and *PM-Apo-acidin4 35S::IYO-GFP* were imaged using mRFP excitation at 561 nm and recording the emitted light at 580 – 650 nm, while SYFP2 or GFP were excited at 488 nm and recorded at 510 – 554 nm. Images were sequentially acquired using a 63x water-immersion objective with a resolution of 1024 x 1024 pixels, unidirectional scan direction, a scan speed of 400 Hz, and a consistent laser power intensity of 10% to prevent cross-channel interference. For z-scans, a pixel size of 0.7 was used.

Raw intensity measurements, without background correction, were obtained for each channel along the plasma membrane of the cells of different cell layers, from the stem cells to differentiated cells, as indicated. The PM-Apo-acidin4 fluorescence ratio was calculated by dividing the SYPF signal (488 nm) by the mRFP signal (561 nm). Nuclear signals corresponded to IYO-GFP. Cell length measurements were taken from the quiescent center and extended to the differentiated cells. Image analysis was performed using the Leica Application Suite software.
