# Supplementary material for: Extracellular pH, cell length and cell differentiation do not firmly correlate across Arabidopsis root tissues
Source: Plant Cell Physiol. 2025 Mar 24;66(6):836–9. doi: 10.1093/pcp/pcaf031 (PMC12290282; doi:10.1093/pcp/pcaf031)
Supplement: pcaf031_Supp [file pcaf031_supp.zip › suppl_data/pcp-2025-e-00010-File004.pdf]

Figure S1.

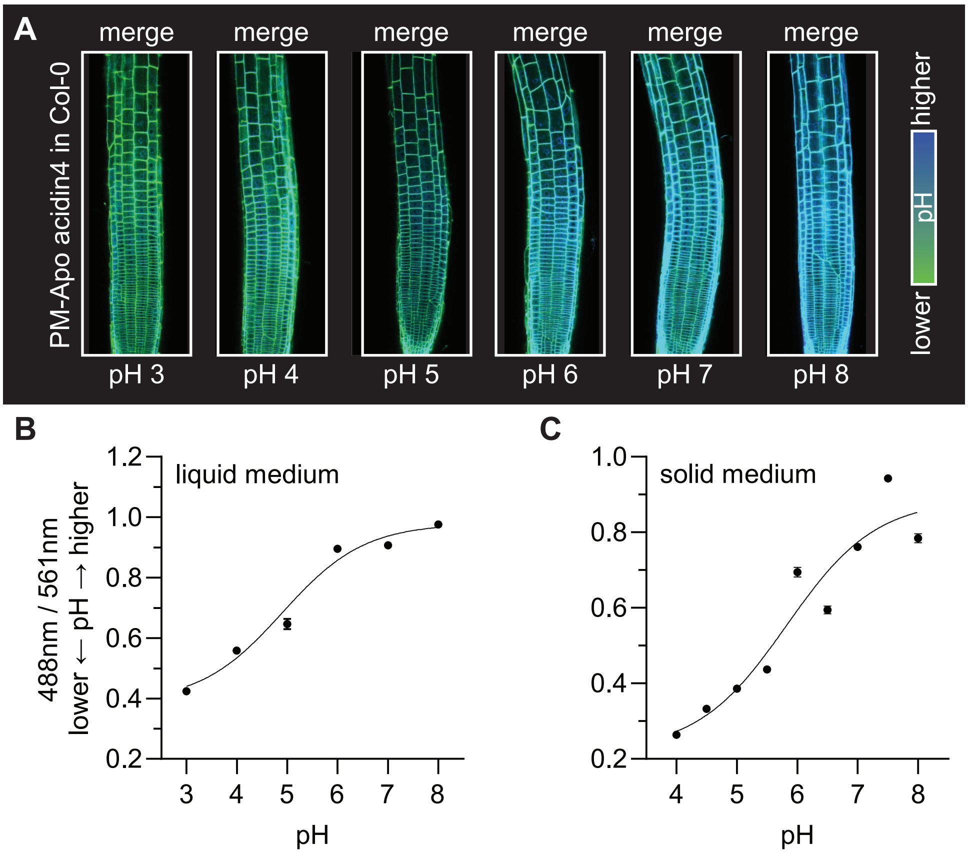

Figure S1. ***In vivo*** **calibration of PM-Apo-acidin4 for extracellular pH estimation in the Arabidopsis root tip.**

(A) Confocal live imaging of 7-day-old *35S::PM-Apo-acidin4* seedlings transferred to liquid MS medium at varying pH levels.

(B-C) *In vivo* calibration curves of PM-Apo-acidin4 fluorescence in Arabidopsis root tips transferred to liquid (B) or solid (C) media at different pH levels. Seedlings were transferred from standard medium (pH 5.7) to liquid or solid MS media adjusted to the indicated pH and imaged as quickly as possible. n= 5 roots and 50 cells per root.
