# Supplementary material for: Extracellular pH, cell length and cell differentiation do not firmly correlate across Arabidopsis root tissues
Source: Plant Cell Physiol. 2025 Mar 24;66(6):836–9. doi: 10.1093/pcp/pcaf031 (PMC12290282; doi:10.1093/pcp/pcaf031)
Supplement: pcaf031_Supp [file pcaf031_supp.zip › suppl_data/pcp-2025-e-00010-File005.pdf]

Figure S2.

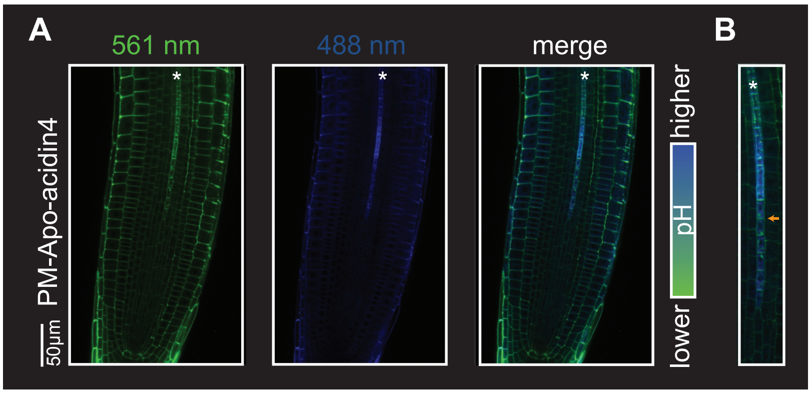

Figure S2. Protophloem sieve elements display higher extracellular pH as they differentiate.

(A) Confocal imaging of 7-day-old seedlings of Arabidopsis roots expressing PM-Apo-acidin4, with asterisks indicating the protophloem cell file.

(B) Close-up on developing protophloem in the merged image of (A), the arrowhead highlights the extracellular signal before the transition to morphologically visible differentiation. Asterisk points out the protophloem cell file.
