# Supplementary material for: Extracellular pH, cell length and cell differentiation do not firmly correlate across Arabidopsis root tissues
Source: Plant Cell Physiol. 2025 Mar 24;66(6):836–9. doi: 10.1093/pcp/pcaf031 (PMC12290282; doi:10.1093/pcp/pcaf031)
Supplement: pcaf031_Supp [file pcaf031_supp.zip › suppl_data/pcp-2025-e-00010-File006.pdf]

Figure S3.

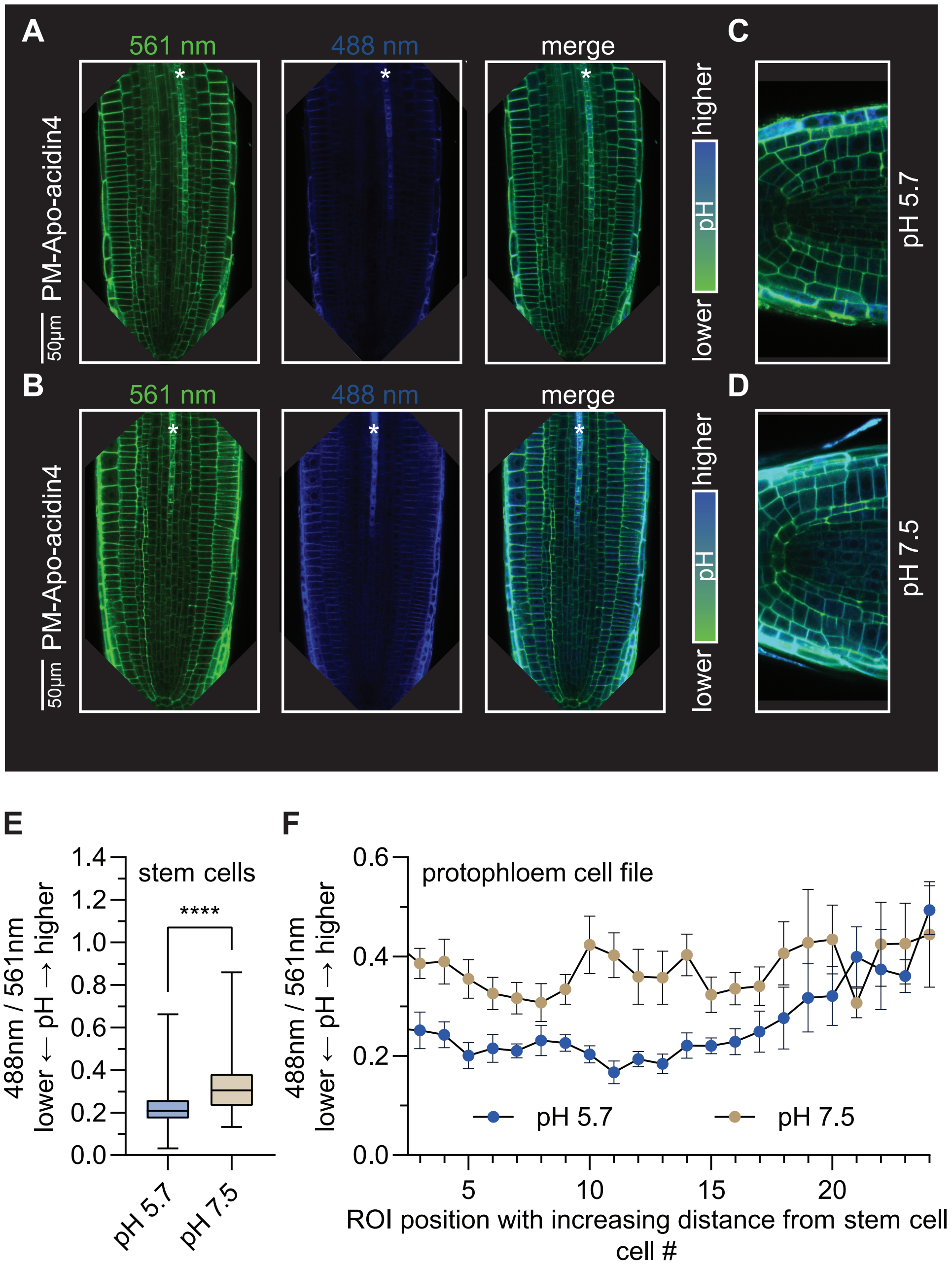

Figure S3. Comparison of extracellular pH (pHₑ) in the stem cell niche and protophloem under varying pH conditions.

(A-B) Confocal live imaging of PM-Apo-acidin4 in the root apical meristem of 7-day-old Col-0 wildtype seedlings, grown on medium at pH 5.7 (A) or pH 7.5 (B), captured at indicated wavelengths. Asterisks mark the protophloem cell file.

(C-D) Close-up views of the stem cell niche in merged images under pH 5.7 (C) and pH 7.5 (D).

(E) Quantification of PM-Apo-acidin4 fluorescence in stem cells of 7-day-old seedlings under standard (pH 5.7) and alkaline (pH 7.5) conditions. Boxplots represent the second and third quartiles and median, with bars indicating maximum and minimum values. n=8 roots with at least 40 cells per root. Statistically significant differences were determined using an unpaired two-tailed t-test. **** p<0.0001.

(F) Ratio-metric quantification of extracellular PM-Apo-acidin4 fluorescence along the protophloem cell files in 7-day-old seedlings grown at pH 5.7 or 7.5. n=8; error bars represent the standard error of the mean.
