# Supplementary material for: Extracellular pH, cell length and cell differentiation do not firmly correlate across Arabidopsis root tissues
Source: Plant Cell Physiol. 2025 Mar 24;66(6):836–9. doi: 10.1093/pcp/pcaf031 (PMC12290282; doi:10.1093/pcp/pcaf031)
Supplement: pcaf031_Supp [file pcaf031_supp.zip › suppl_data/pcp-2025-e-00010-File007.pdf]

Figure S4.

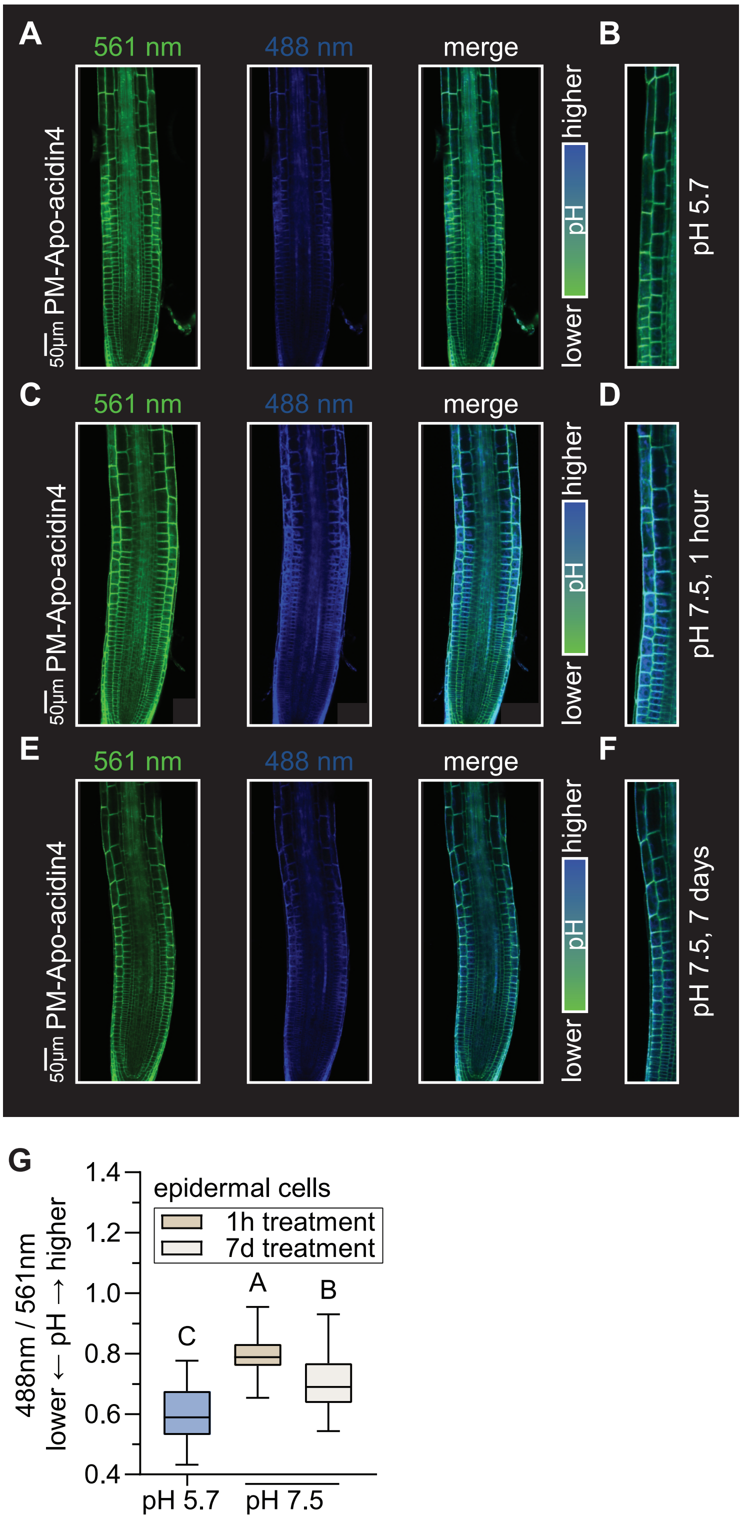

Figure S4. Dynamics of root extracellular pH following transfer to alkaline media.

(A-F) Confocal live imaging of PM-Apo-acidin4 in 7-day-old seedlings under different medium conditions: standard medium (pH 5.7) (A-B), medium shifted to pH 7.5 for 1 hour (C-D), and continuous growth on alkaline medium (pH 7.5) (E-F). Panels B, D, and F show close-up views of the epidermis in A, C, and E, respectively.

(G) Quantification of PM-Apo-acidin4 fluorescence in the epidermis under both standard pH (5.7) and alkaline pH (7.5) mediums. Alkaline treatments were performed for 1 hour or 7 days. n=5 roots and 150 cells per root. Box plots display 2nd and 3rd quartiles and the median, whiskers indicate maximum and minimum. Statistical significance was determined by two-way ANOVA with Tukey's test. $p<0.0001$ in pairwise comparison.
