# Supplementary material for: Extracellular pH, cell length and cell differentiation do not firmly correlate across Arabidopsis root tissues
Source: Plant Cell Physiol. 2025 Mar 24;66(6):836–9. doi: 10.1093/pcp/pcaf031 (PMC12290282; doi:10.1093/pcp/pcaf031)
Supplement: pcaf031_Supp [file pcaf031_supp.zip › suppl_data/pcp-2025-e-00010-File008.pdf]

Figure S5.

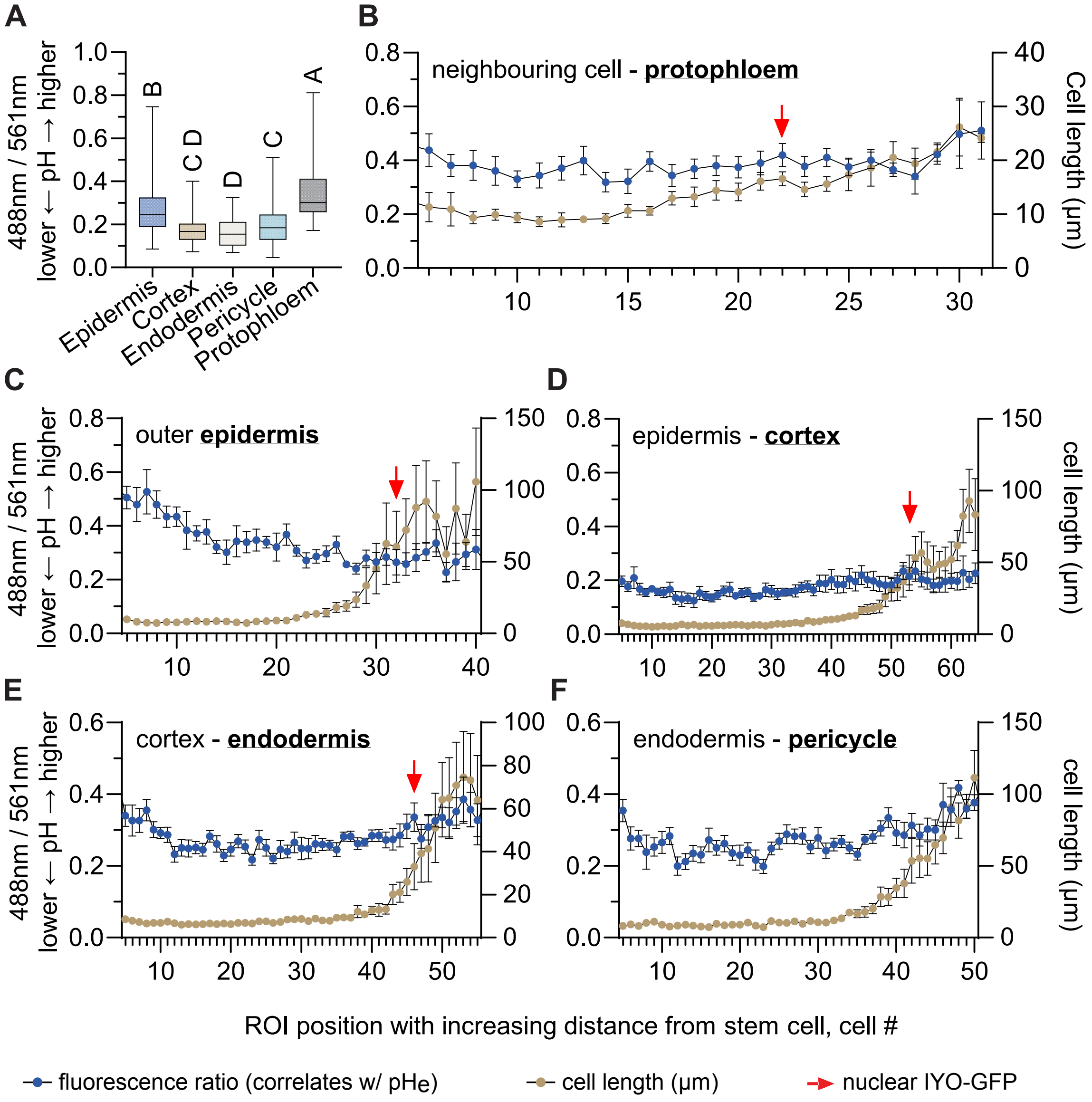

Figure S5. Periclinal extracellular pH analysis across multiple root tissues during elongation and differentiation.

(A) Ratio-metric quantification of PM-Apo-acidin4 fluorescence in optical cross sections of Arabidopsis roots. Measurements were taken across all cell layers at the location where protophloem differentiation begins. n=12. Box plots display 2nd and 3rd quartiles and the median, whiskers indicate maximum and minimum. Statistical differences were determined using one-way ANOVA followed by Tukey's test. $p<0.0021$ for pairwise comparison.

(B-F) Quantification of periclinal PM-Apo-acidin4 fluorescence, cell length, and IYO-GFP nuclear accumulation at specific tissue boundaries: neighboring cell – **protophloem** (B), outer **epidermis** (C), epidermis – **cortex** (D), cortex – **endodermis** (E), and endodermis – **pericycle** (F). Tissues in bold and underline were used for cell length measurements. n=8. Error bars indicate standard error of the mean.
