# Supplementary material for: Extracellular pH, cell length and cell differentiation do not firmly correlate across Arabidopsis root tissues
Source: Plant Cell Physiol. 2025 Mar 24;66(6):836–9. doi: 10.1093/pcp/pcaf031 (PMC12290282; doi:10.1093/pcp/pcaf031)
Supplement: pcaf031_Supp [file pcaf031_supp.zip › suppl_data/pcp-2025-e-00010-File009.pdf]

Figure S6.

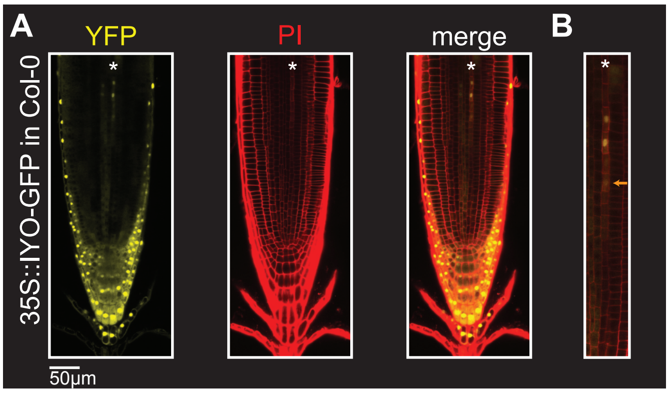

Figure S6. Nuclear accumulation of IYO-GFP in Arabidopsis root tips.

(A) Confocal microscopy images of IYO-GFP with propidium iodide (PI) counterstaining in the root apical meristem of *35S::IYO-GFP* 7-day-old seedlings. Asterisks indicate the protophloem cell files.

(B) Close-up on developing protophloem in the image of (Alength), the arrowhead highlights the appearance of the GFP nuclear signal. Asterisk points out the protophloem cell files.
